# Time Course of the Changes in Novel Trioxane Antimalarial 99/411 Pharmacokinetics upon Antiepileptic Drugs Co-Administration in SD Rats

**DOI:** 10.1155/2014/756965

**Published:** 2014-10-14

**Authors:** Yeshwant Singh, Hari Narayan Kushwaha, Anamika Misra, Mahendra Kumar Hidau, Shio Kumar Singh

**Affiliations:** ^1^Pharmacokinetics & Metabolism Division, CSIR-Central Drug Research Institute, Sector-10, Jankipuram Extension, Sitapur Road, Lucknow 226031, India; ^2^Academy of Scientific and Innovative Research, New Delhi 110025, India

## Abstract

*Objective*. The study aimed to evaluate the influences of coadministration of antiepileptic drugs (AEDs) on an antimalarial candidate 99/411 pharmacokinetic (PK) profile. *Method*. For this, single oral dose PK drug interaction studies were conducted between 99/411 and FDA approved AEDs, namely, Phenytoin (PHT), Carbamazepine (CBZ), and Gabapentin (GB) in both male and female SD rats, to assess the coadministered and intersexual influences on 99/411 PK profile. *Results*. Studies revealed that there were no significant alterations in the PK profile of 99/411 upon PHT and CBZ coadministration in both male and female rats, while systemic exposure of 99/411 was significantly increased by about 80% in female rats upon GB coadministration. In terms of AUC, there was an increase from 2471 ± 586 to 4560 ± 1396 ng*·*h/mL. Overall, it was concluded that simultaneous administration of AEDs with 99/411 excludes the requirements for dose adjustment, additional therapeutic monitoring, contraindication to concomitant use, and/or other measures to mitigate risk, except for GB coadministration in females. These findings are further helpful to predict such interactions in humans, when potentially applied through proper allometric scaling to extrapolate the data.

## 1. Introduction

In a number of clinical situations, concomitant use of two or more drugs is imperative. In particular in chronic disease treatment course, patients must take more than one drug for an extended period of time. In this course, many drug interactions have received a great deal of attention to avoid any side effects to the patients. The greater the number of drugs prescribed to a patient, the greater the risk of drug interactions. In an epidemiological study, conducted at a community hospital to investigate the clinical relevance of drug-drug interactions (DDIs), it was reported that incidence of interactions raises up to 7% in those patients taking 6–10 drugs and 40% in those taking 16–20 drugs a day [1]. Beside the off target effects, DDIs can lead to termination of drug development in early stages, refusal of approval, very strong prescribing restrictions, and withdrawal of drugs from market [2]. Furthermore, frequent and severe side effects due to DDIs in elderly patients because of the impaired physiological functions are reportedly increasing, necessitating the requirement of more information regarding the DDIs, while giving any combination therapy [3].

Many of the major pharmacokinetic (PK) interactions between drugs are due to cytochrome 450 (CYP450) enzymes, whose genetic expressions are being affected (induction or inhibition) by previously or simultaneously administered drugs. In addition, such interactions may exist at the level of absorption, distribution, clearance, and transporters. Such interactions are investigated in terms of the PK parameter changes of the concerned drug. These substantial changes in PK parameters may be used to indicate clinical importance of DDIs, when substrate and interacting drugs are likely to be given simultaneously either for short periods or chronically for an extended periods of time during the combinational therapy.

The present study aimed to evaluate the influences of coadministration of antiepileptic drugs (AEDs) on an antimalarial candidate 99/411 pharmacokinetic (PK) profile. 99/411 is a novel 1, 2, 4 trioxane derivative, antimalarial drug candidate with peroxide scaffold essential for its pharmacological activity [4, 5]. This molecule has currently entered into phase I clinical trial. During the drug developmental stages,* in vivo* drug interaction studies are usually carried in experimental animals because of the difficulties to conduct these studies in humans at this stage [6, 7]. These studies are further helpful in predicting the drug interactions in human beings, although results obtained in the experimental animals do not ensures the existence of the same in humans [8], provided extrapolation of these findings is carefully done by considering physiochemical variables in higher species as an important function of the body weight across the species.

In this study, our investigational drug 99/411 was considered as a substrate and the interacting agents chosen were FDA approved antiepileptic drugs (AEDs) including Phenytoin (PHT), Carbamazepine (CBZ), and Gabapentin (GB). The rationale behind selecting AEDs as interacting drugs is being strengthened by the fact that the seizures (characteristics of epilepsy) are one among the common feature of malaria regardless of its severity [9]. Seizures are found to occur in about 85% of the cases diagnosed of cerebral malaria [10, 11]. Thus the coexistence of these two diseases leads to a combinational therapy involving simultaneous administration of AEDs and antimalarials, making the importance of investigating DDIs.

## 2. Material and Method

### 2.1. Chemicals and Reagents

**Figure 1:**
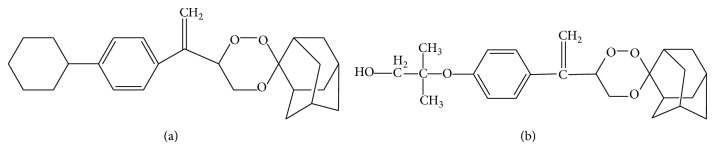
Chemical structure of 99/411 (a) and 97/63 (b).

 Pure (>99%) reference standards of 99/411 and 97/63 (used as IS) (Figures 1(a) and 1(b)) were obtained from Medicinal & Process Chemistry Division, CSIR-Central Drug Research Institute (CDRI), Lucknow, India. Acetonitrile, HPLC grade, was purchased from Thomas Baker (Chemicals) Limited (Mumbai, India). Analytical grade ammonium acetate for buffer preparation was obtained from SD Fine-Chemicals Limited (Mumbai, India). Glacial acetic acid was procured from E Merck (India). Ultrapure water was obtained from a Milli-Q PLUS PF system. AEDs were obtained as gift samples. PHT and CBZ were obtained from Ranbaxy Research Laboratories (Gurgaon, India). GB was obtained from Lupin Limited Research Park (Pune, India). Drug free rat plasma was collected from healthy male and female* Sprague-Dawley* (SD) rats provided by National Laboratory Animal Centre (NLAC), CDRI.

### 2.2. Subjects

Healthy male and female SD rats obtained from NLAC of CDRI, India, were weighed 200–250 g on the day of dosing. Prior to the studies, animals were acclimatized for 4 days in proper ventilated polypropylene cases in standard laboratory conditions with regular 12 h light-dark cycle, temperature (22 ± 2°C), and relative humidity (55 ± 5%). Standard pellet laboratory chow and water were allowed ad lib. Guidelines approved by the “Animal Experimentation Ethics Committee” and Good laboratory practice (GLP) were followed throughout the animal experimentation.

### 2.3. Design

The studies were single dose oral PK, designed to estimate the effect of concurrent administration of AEDs on PK profile of 99/411. Individual studies were conducted for each AED coadministration with 99/411. Each study was carried out in two groups of experimental animals, every group comprising three animals (*n* = 3). Studies were conducted separately on male and female SD rats to assess the intersex differences in the drug interaction pattern. Subjects were given 12 mg/Kg of 99/411 (control) and 99/411 followed by coadministration of AEDs (42 mg/Kg each) in separate sets of experimental animals. Blood samples for PK analysis were collected before dosing and at the following time points 0.083, 0.33, 0.75, 1, 1.5, 2, 4, 6, 8, 12, 24, 36, 48, 60, 72, and 96 hours after dose.

### 2.4. Formulation

Fresh oral formulations of 99/411 and each AEDs were prepared in neutralized arachis oil (as a suspension) at 12 and 42 mg/Kg dose, respectively. Both the formulations were subjected to quality control (QC), stability, and homogeneity test to ensure the strength before dosing. The volume factor for both the formulations was 1 mL/Kg.

### 2.5. Bioanalysis

#### 2.5.1. Sample Analysis

Blood samples collected for quantitative estimation of 99/411 were processed by centrifugation within 30 min of sample collection to obtain plasma and were stored at −70°C till analysis. 10 *µ*L of IS was spiked into each study samples followed by a double step liquid-liquid extraction procedure using in n-hexane to extract the drugs and IS. Plasma concentration was determined by using a fully validated LC-MS/MS method. Mass spectrometric detection was performed on an API 4000 LC-MS/MS mass spectrometer (Applied Biosystems, MDS Sciex, USA) with Analyst 1.4 software. Product ion transitions (ammonium adducts of analyte and IS), at* m/z* 412.3 to 185.1 and 418.2 to 119.1 were used for quantification of 99/411 and IS respectively. The assay was linear over the range 1.56–200 ng/mL with LOQ 1.56 ng/mL [5]. Coefficients of determination (*r* ^2^) were >0.99 for standard curves generated. Precision and accuracy of the method was determined by analyzing QCs at 1.50, 80 and 160 ng/mL. All accuracies were within 15% of the nominal concentrations with standard deviation of <8%.

#### 2.5.2. Pharmacokinetic and Statistical Analysis

The primary endpoints for these studies were area under the curve (AUC_0–*∞*_), maximum plasma concentration (*C*_max⁡_), time to attain *C*_max⁡_ (*T*_max⁡_), elimination half-life (*T*_1/2_), mean residence time (MRT), and relative bioavailability (RB), when 99/411 was given alone and coadministered with AEDs in male and female SD rats. These PK parameters were calculated by noncompartmental analysis using Winnonlin software (Version 1.5, Pharsight Corporation). The estimated PK parameters for 99/411 alone and coadministered with AEDs were statistically compared using two-tailed Student's* t*-test. In all the tests, a probability level of significance was kept at *α* = 0.05. Results were expressed as mean ± SD. RB was calculated as

(1) $$\begin{array}{c} \text{Relative}\;\text{bioavailability}\left(\text{RB}\right)=\frac{{\text{AUC}}_{\text{Co-admin}}}{{\text{AUC}}_{\text{Control}}}\times 100. \end{array}$$

## 3. Results

### 3.1. 99/411 Pharmacokinetics in Male and Female SD Rats

**Figure 2:**
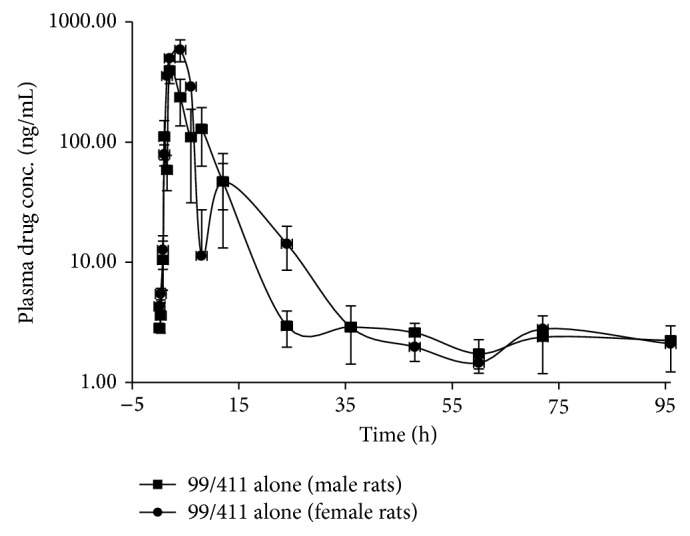
Plasma concentration-time profile of 99/411 alone in male and female rats.

To evaluate the coadministered influences of AEDs on PK profile of 99/411, its baseline PK profile was generated at 12 mg/Kg in healthy male and female rats. After oral dose administration, AUC_0–*∞*_ values were found to be higher in female rats in comparison to male rats, but with no statistical significant difference (Table 1). PK parameters of 99/411 alone in male and female rats were compared statistically by “Student's* t*-test: two-sample assuming unequal variances.” Statistically no significant difference was observed in *T*_1/2_, *T*_max⁡_, *C*_max⁡_, AUC_0–*∞*_, and MRT in terms of *P* value (*P* > 0.05). The plasma concentration-time profiles (mean ± SD, *n* = 3) for 99/411 in male and female rats have been shown in Figure 2.

### 3.2. 99/411 upon Coadministration with PHT in Male and Female Rats

In male rats, mean values of *T*_1/2_, *T*_max⁡_, *C*_max⁡_, AUC_0–*∞*_, and MRT for 99/411 upon coadministration of 99/411 with PHT were 2.10 ± 0.30 h, 7.00 ± 1.00 h, 489.00 ± 166.4 ng/mL, 2521.93 ± 619.89 ng*·*h/mL, and 17.58 ± 2.85 h, while for female rats values were 4.65 ± 0.67 h, 4.00 ± 1.18 h, 493.85 ± 73.14 ng/mL, 2160.70 ± 488.58 ng*·*h/mL, and 12.04 ± 1.00 h, respectively. PK profiles after oral coadministration of 99/411 with PHT have been given in Table 1.

### 3.3. Intersex Comparison

**Figure 3:**
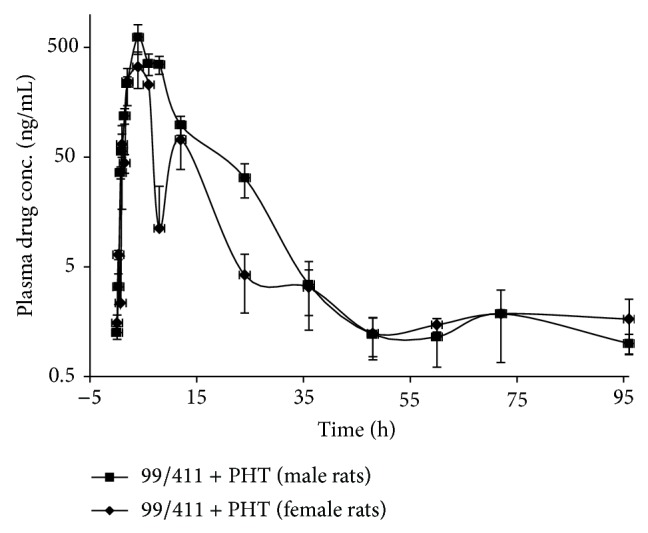
Plasma concentration-time profile of 99/411 upon Phenytoin coadministration in male and female rats.

Intersex statistical analysis of PK parameters for 99/411 upon coadministration with PHT in male and female rats revealed that no significant difference existed between male and female PK profiles in terms of *P* values which were found >0.05 for respective parameter. However, RB was found to be enhanced in male rats, while a decrease was seen in female rats (Table 1). The plasma concentration-time profiles (mean ± SD, *n* = 3) for 99/411 upon PHT coadministration in male and female rats have been shown in Figure 3.

### 3.4. 99/411 upon Coadministration with CBZ in Male and Female Rats

Mean values of *T*_1/2_, *T*_max⁡_, *C*_max⁡_, AUC_0–*∞*_, and MRT for 99/411 upon coadministration of 99/411 with CBZ were 3.16 ± 0.49 h, 6.50 ± 0.50 h, 495.25 ± 58.42 ng/mL, 2534.84 ± 299.69 ng*·*h/mL, and 13.84 ± 1.56 h in male rats, while 3.49 ± 1.09 h, 4.00 ± 1.18 h, 484.74 ± 60.50 ng/mL, 2095.20 ± 298.73 ng*·*h/mL, and 12.17 ± 0.98 h for female rats, respectively (Table 1). PK profiles after oral coadministration of 99/411 with CBZ have been given in Table 1.

### 3.5. Intersex Comparison

**Figure 4:**
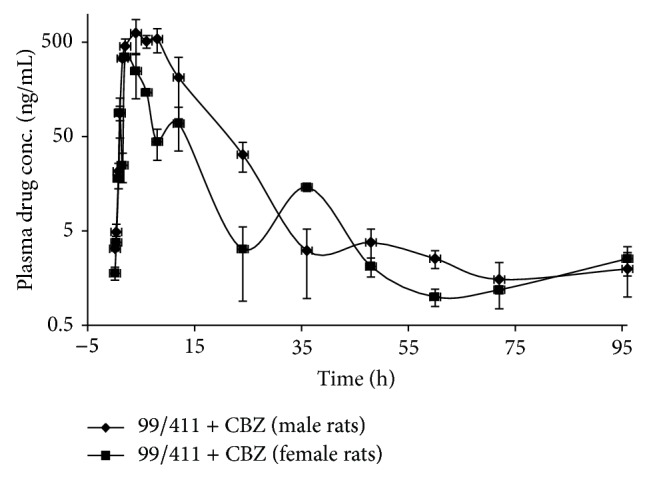
Plasma concentration-time profile of 99/411 upon Carbamazepine coadministration in male and female rats.

Intersex statistical analysis of PK parameters for 99/411 upon coadministration with CBZ in male and female rats revealed that no significant difference existed between male and female PK profiles in terms of *P* values. However, like PHT coadministration, RB was found to be enhanced in male rats, while a decrease was seen in female rats (Table 1). The plasma concentration-time profiles (mean ± SD, *n* = 3) for 99/411 upon CBZ coadministration in male and female rats have been shown in Figure 4.

### 3.6. 99/411 upon Coadministration with GB in Male and Female Rats

Mean values of *T*_1/2_, *T*_max⁡_, *C*_max⁡_, AUC_0–*∞*_, and MRT for 99/411 upon coadministration of 99/411 with GB were 3.16 ± 0.49 h, 6.50 ± 0.50 h, 495.25 ± 58.42 ng/mL, 2534.84 ± 299.69 ng*·*h/mL, and 13.84 ± 1.56 h in male rats, while 5.02 ± 1.09 h, 2.00 ± 1.11 h, 449.64 ± 77.95 ng/mL, 4560.86 ± 1396.05 ng*·*h/mL, and 14.05 ± 1.56 h for female rats, respectively. PK profiles after oral coadministration of 99/411 with GB have been given in Table 1.

### 3.7. Intersex Comparison

**Figure 5:**
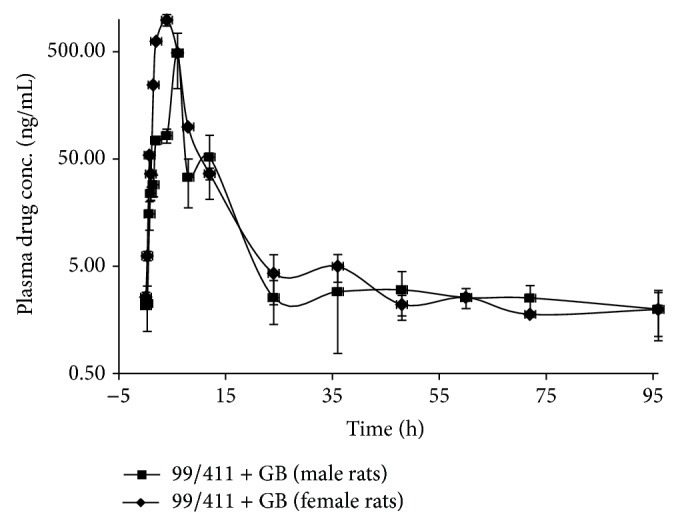
Plasma concentration-time profile of 99/411 upon Gabapentin coadministration in male and female rats.

Intersex statistical analysis of PK parameters for 99/411 upon coadministration of 99/411 with GB in male and female rats revealed that no significant difference existed between male and female PK profiles in terms of *P* values. However, RB was found to be decreased in male rats, while an increase was seen in female rats (Table 1). The plasma concentration-time profiles (mean ± SD, *n* = 3) for 99/411 upon GB coadministration in male and female rats have been shown in Figure 5.

## 4. Discussion

This study was conducted to evaluate the influence of concurrent administration of 99/411 with FDA approved AEDs. Keeping in view, the effect of species and sex differences on the PK profile of concerned drugs, study was conducted in healthy male and female SD rats [12, 13]. First of all, the baseline PK profile was generated in male and female rats. Due to fluctuating plasma concentration-time profile (Figure 2) the use of noncompartmental analysis was preferred. Therefore, PK parameters of 99/411 were obtained by noncompartmental analysis and are listed in Table 1.

PHT, one of the frequently used AED, is well known to lower the plasma levels of a number of drugs, which reduces therapeutic efficacy of concurrently administered drugs [14]. Study was conducted to evaluate the influence of PHT on PK profile of 99/411. Statistical comparison of PK parameters of 99/411 have shown no significance difference (in terms of *P* values > 0.05) from baseline PK profile, indicating that PHT coadministration has little influence on PK parameters of 99/411. RB of 99/411 was observed to be enhanced by about 20% in male rats, while a decrease of about 13% was found in female rats. When PK profiles of male and female rats were statistically compared to evaluate intersex effect, it was observed that significance difference existed for *T*_1/2_ values, while for other PK parameters no significance difference existed (Table 1). Although individual differences in the expression of drug transporters along GIT and intersex hormonal differences may be responsible for the differences in the RB, but in context to this study, the alteration in RB values is due to individual variations rather than any realistic differences among male and female rats. However, such differences in pharmacokinetics and pharmacodynamics due to gender differences are well reported in animals and humans. Gender is one among the influential variable that contributes significantly to differences in pharmacokinetics including absorption, distribution, metabolism, and excretion [15].

Similar to PHT, CBZ is one of most frequently prescribed AED. Its widespread and long term use with other medications enhance the possibilities of drug interactions. Study was carried out to evaluate the influence of CBZ on PK profile of 99/411. It was found that RB of 99/411 was enhanced by about 20% in male rats, while a decrease of about 15% was found in female rats, similar to that of 99/411 coadministration with PHT, but statistically nonsignificant. No significant differences were observed too for other PK parameters of 99/411 in both the sexes.

Like PHT and CBZ, GB is widely used AED, which possesses quite desirable PK properties. It does not bind to plasma proteins nor is metabolized and does not induce/inhibit liver enzymes, reducing the likelihood of drug interaction with other drugs. Although this drug is rapidly absorbed in small intestine by carrier mediated transport system, which is saturable [16]. This leads to the possibility of DDI in the absorption phase of 99/411. In an* in situ* single pass intestinal perfusion study, intestinal transporters have been reported to play a role in 99/411 absorption [17]. Study was conducted to evaluate GB coadministration on 99/411 PK profile. Study revealed that there was slight decrease in the systemic exposure (AUC_0–*∞*_) of 99/411 in male rats, while an increased AUC_0–*∞*_ was found for female rats. In terms of RB, RB of 99/411 was significantly increased by about 80% in female rats, while no effect was observed in male rats. Thus, a significant difference was observed intersexually in the interaction pattern influencing the RBs. The RB was almost unaltered in male rats, while significantly enhanced in female rats. In male rats, there was a shift in *T*_max⁡_ from 2 ± 0.00 to 6.50 ± 0.50 h, indicating that GB coadministration interacted at absorption level, which might have reduced absorption rate of 99/411; however, systemic exposure was unaltered. Conversely, in female rats, *T*_max⁡_ was shifted from 4.50 ± 1.53 to 2.00 ± 1.11 indicating fast absorption of 99/411 upon GB coadministration which might have resulted into enhanced systemic exposure of 99/411.

## 5. Conclusion

Overall, these studies explicitly indicated that coadministration of these AEDs does not exert any significant effect on PK of 99/411, except for GB coadministration in female rats. A similarity was observed in the interaction pattern of PHT and CBZ, where an increase in the RB of 99/411 was reported in male rats, while a decrease in female rats, which was nonsignificant though. In contrast, opposite pattern was found for GB coadministration, where a significant increase of about 80% was reported in female rats, while no effect was reported in male rats. However, these studies were conducted on a randomized selection basis of animal group (*n* = 3); results represented herein do not negate the variations in the interaction pattern. These findings could not be directly utilized to estimate and predict existence of similar interactions in humans, unless predictions are not rationalized through allometric scaling approaches, keeping in view all the anatomical, physical, and chemical variations across species.

## Figures and Tables

**Table 1:** 99/411 pharmacokinetics, alone and upon coadministration with AEDs in SD rats [data expressed as mean ± SD (*n* = 3)].

PK parameters	99/411 alone		99/411 + GB		99/411 + CBZ		99/411 + PHT	
	Male	Female	Male	Female	Male	Female	Male	Female
*C*_max⁡_ (ng/mL)	379.25 ± 32.25	561.25 ± 195.27	484.50 ± 127.40	449.64 ± 77.95	495.25 ± 58.42	484.74 ± 60.50	489.00 ± 166.4	493.85 ± 73.14
*T*_max⁡_ (h)	2.00 ± 0.00	4.50 ± 1.53	6.50 ± 0.50∗	2.00 ± 1.11∗	6.50 ± 0.50∗	4.00 ± 1.18	7.00 ± 1.00	4.00 ± 1.18
AUC_0–*∞*_ (ng*·*h/mL)	2124 ± 317	2471 ± 586	1981 ± 471	4560 ± 1396∗	2544 ± 299	2095 ± 298	2582 ± 619	2160 ± 488
*T*_1/2_ (h)	3.33 ± 0.36	3.97 ± 0.59	2.66 ± 0.65∗	5.02 ± 1.09∗	3.16 ± 0.49	3.49 ± 1.09	2.10 ± 0.30∗	4.65 ± 0.67
MRT (h)	10.56 ± 0.38	8.94 ± 1.02	13.46 ± 2.35	14.05 ± 1.56	13.84 ± 1.56	12.17 ± 0.98	17.58 ± 2.85	12.04 ± 1.00
RB^#^ (%)	—	—	93	184∗	119	85∗	121∗	87

^*^Values are statistically significantly different at 95% CI (*P* < 0.05); ^#^relative bioavailability.
